# Experimental Investigation and Performance Optimization during Machining of Hastelloy C-276 Using Green Lubricants

**DOI:** 10.3390/ma15155451

**Published:** 2022-08-08

**Authors:** Gurpreet Singh, Vivek Aggarwal, Sehijpal Singh, Balkar Singh, Shubham Sharma, Jujhar Singh, Changhe Li, R.A. Ilyas, Abdullah Mohamed

**Affiliations:** 1 Department of Mechanical Engineering, IKGPTU Main Campus, Kapurthala 144603, Punjab, India; 2 Department of Mechanical Engineering, Guru Nanak Dev Engineering College, Ludhiana 141006, Punjab, India; 3 Department of Mechanical Engineering, University Centre for Research and Development, Chandigarh University, Mohali 140413, Punjab, India; 4 Department of Mechanical Engineering, Indian Institute of Technology, IIT-Ropar, Rupnagar 140001, Punjab, India; 5 School of Mechanical and Automotive Engineering, Qingdao University of Technology, Qingdao 266520, China; 6 School of Chemical and Energy Engineering, Faculty of Engineering, Universiti Teknologi Malaysia, Johor Bahru 81310, Johor, Malaysia; 7 Centre for Advanced Composite Materials, Universiti Teknologi Malaysia, Johor Bahru 81310, Johor, Malaysia; 8 Research Centre, Future University in Egypt, New Cairo 11835, Egypt

**Keywords:** Hastelloy C-276, minimum quantity lubrication, sustainable development goals, response surface methodology, vegetable oil, synthetic oil, waste oil, SEM

## Abstract

Smart manufacturing is the demand of industry 4.0, in which the mass production of difficult-to-cut materials is of great concern to fulfil the goal of sustainable machining. Presently, the machining of superalloy is of upmost interest because of its wide application. However, the limited data on the turning of Hastelloy C-276 highlights its challenges during processing. Hence, the machining performance of superalloy considering surface quality, thermal aspects and chip reduction coefficient was examined with minimum quantity lubrication of several oils to address the sustainable development goal (SDG-12). The output responses were optimized through response surface methodology along with analysis of variance. The research exhibited that the output responses were dominated by cutting speed and feed rate having a percentage benefaction of 24.26% and 60%, respectively, whilst the depth of cut and lubricant type have an influence of 10–12%. No major difference in temperature range was reported during the different lubrication conditions. However, a substantial variation in surface roughness and the chip reduction coefficient was revealed. The percentage error evaluated in surface roughness, temperature and chip reduction coefficient was less than 5%, along with an overall desirability of 0.88, describing the usefulness of the model used. The SEM micrograph indicated a loss of coating, nose and flank wear during all lubrication conditions. Lastly, incorporating a circular economy has reduced the economic, ecological and environmental burden.

## 1. Introduction

The machining of Hastelloy C-276 is quite difficult during dry machining conditions because of its low thermal conductivity. Nevertheless, its excellent strength along with a high heat carrying capacity makes it suitable for components of air craft, petro and chemical plants’ check valves as well as nuclear reactor components. In addition, the nickel-based alloy sustains its mechanical strength, creep and resistance to wear at elevated temperatures, making it suitable for aerospace and other fields [1,2,3,4,5]. The input variables have a direct impact on the performance of nickel alloy, so the correct identification of input variables is significant during the metal cutting of super alloys [6]. An investigation into the machining of Hastelloy C-2000 explored the idea that increments of cutting speed along with the feed rate impacts the surface quality of a machined product as well as the forces during cutting [7]. For the enhancement of machining performance, the utility of cutting fluids and coolant are required for machining super alloys and leads to a larger heat evolution, progression of built up edge, chip clinging and fast tool wear that further degrade the workpiece quality. Therefore, the use of an efficient and environmentally friendly lubricant is always needed to enhance the product’s quality and tool life [8,9]. The use of the traditional cooling system, as well as its poor handling and care, has resulted in ecological imbalance, health hazards, corrosion, fumes and chemical reactions as well as the addition of extra cost to manufacturing. Additionally, the expense of recycling equipment, wastage disposal and the procurement costs of petroleum-based products is a major issue against sustainable machining. Hence, suitable cooling systems like MQL and other chilling media are of major interest [10,11]. The mechanical cutting of super alloy is performed these days with various low-temperature cooling techniques to obtain better machining output in the form of surface quality, cutting forces, chip control and power consumption [12,13,14,15]. Research on the machining of Inconel 825 without lubricant usage reported the thermal cracking and sheared-edge flow of work metal. However, the application of cryogenic cooling during the milling of Ni-Cr alloys reduced the chip serration along with control over tool damage [16,17]. Investigations on tool damage during the processing of super alloys reported that abrasion, adhesion, diffusion and notch wear was observed during the processing of Ni alloys [18]. A wear examination of the cutting insert found that the scratches on the tool flank appear while raising the cutting velocity because of the material constituents’ abrasive elements. Further, this leads to chip sticking on the top face of tools at larger revolutions due to a higher heat generation [19]. The turning performance of difficult-to-cut materials examined during flood and dry machining exhibited that an increasing feed rate generates more friction and plastic deformation at the shear section which results in the higher cutting forces of the dry machining system [20,21]. The machining output of processing the C-276 super alloy using low-temperature cooling reported an appreciable minimization of heat generation, surface roughness, cutting forces and tool damage compared to other conditions [22]. A performance examination on the turning of UNS S15500 utilizing MQL and modelling with response surface methodology (RSM) accompanying the central composite design (CCD) techniques exhibited identical results among predicted and actual responses. Along with this, the flow rate of MQL plays an important role in enhancing the surface quality and tool life [23]. An investigation during the machining of D_2_, UNS 17400-SS under dry and MQL conditions reported a low temperature, good surface quality and larger insert expectancy in contrast to dry conditions [24,25]. To minimize the number of experiments, cost, time and adequate modelling, the application of design of experiment (DOE) is recommended, which can facilitate the plan of experiments, model formulation and optimization through RSM, Taguchi and full factorial design [26,27]. Henceforth, in the current study, RSM was executed along with the CCD model for the conduct of the experiment, data modelling and the optimization of input variables. This technique has been successfully applied for the mathematical modelling of Titanium alloy and revealed that the output responses were close to predicted values [28]. Further, the implementation of this statistical approach for the modelling of SR, tool wear and material removal rate (MRR) during the cryo-machining of 17-4 SS steel using an L-20 RSM-based CCD technique revealed that the predicted results and experimental outputs were within close range [29]. A critical review on the ecological, economic and technological prospective suggested that eco-friendly machining is the need of the present industrial scenario which can be possible through the utilization of sustainable cooling technologies [30]. Experimental investigations on super alloy C-276 with a cermet insert and ANOVA optimization found optimum conditions at the lowest level of input variables; along with this, surface quality and tool wear was also investigated at different levels of (speed) v, (feed) f and (depth of cut) doc [31]. Investigations on the forces, SR and tool damage during the machining of Inconel 800 alloy utilizing vegetable-based oil amalgamated with aluminum oxide nano particles employed ANOVA analysis, a CDA approach, Box–Cox plot and normal probability graph and concluded that vegetable-based nano-fluid MQL cooling enhanced machining performance [32]. Examinations on the difficult to cut materials require low temperature cooling system to enhance machining capability and the input variable have impact on the performance [33,34,35,36,37]. Research on eco-friendly cutting fluids for sustainable cooling techniques suggested the use of the MQL system along with vegetable-based oil, nano fluids and cryogenic cooling as an alternative to the conventional cooling system. In addition to this, various types of vegetable oils used for machining were listed as coconut oil, groundnut oil, palm oil, kernel oil, soybean oil, castor oil, olive oil and sunflower oil. Further, the cause of superior lubrication and cooling action of these oils has also been mentioned due to their fatty acid chains, polar molecules, London dispersion force and high viscosity. Moreover, it was also mentioned that using nano-MQL for processing of difficult-to-cut materials would give a better performance. The major challenges to the practical execution of a cryogenic approach on the shop floor are the non-availability of standard equipment, freezing of machining systems, higher initial setup, inability to recycle cryogenic fluid, higher consumption of cryogenic liquid, larger machining cost and the requirement of special knowledge [38,39,40,41]. Despite the practical limitations, cryogenic cooling has environmentally friendly behavior compared to the conventional cooling system [42]. However, along with the several advantages of cryogenic cooling, it suffers in many aspects during the machining of super alloy and ferrous materials. The increase in workpiece hardness, higher cutting forces, microstructure change and cold cracking are the main drawbacks of low-temperature cooling, and all these factors divert the attention of researchers toward the different types MQL. In addition to this, oil-based cooling is still the best option for the machining of ferrous and super alloys compared to cryogenic cooling [39]. An investigation on the drilling of lighter alloys AZ91 revealed the influence of input parameter on machining performance [43,44]. Moreover, the various nano-fluids mixed with vegetable oil used for MQL were enlisted as Al_2_O_3_, MoS_2_, SiO_2_, CNT, Nanoplatelet and ZrO_2_ [43,44,45]. From the literature study, it has been concluded that vegetable-based oil MQL has a potential as a sustainable cooling technique for machining the different categories of ferrous and other materials. The utility of conventional lubrication in the present industrial scenario is challenging due to the environmental consciousness, strict environmental guidelines and adverse effects of petroleum-based cutting fluids. It leads to more consumption of power, wastage disposal and overall machining cost. Also, it releases the chemical waste and carbon emissions as well as exposure to occupational diseases [46,47,48,49,50,51,52].

### 1.1. Significance of the Study

The lubricants which have minor negative impacts on health, the environment and machining performance are referred to as green lubricants. The significance of the present study focuses on the performance capability of synthetic, vegetable and waste oils for machining Ni-based alloy in terms of the surface roughness, temperature and chip reduction coefficient. Further, the utility of different oils and MQL machining in line with UN sustainable development goal (SDG-12) has been studied. In addition, an outlook on the global scenario of the lubricant budget and their consumption impacting the reserve of non-renewable energy resources, environment degradation and the capital of the enterprise has also been included. Moreover, the investigation on the intensity of tool wear in different lubricant signifies the economic as well as ecological prospective of MQL for machining super alloys, which is rarely available in the literature.

#### 1.1.1. Scope of Sustainable Development Goal (SDG-12)

**Figure 1:**
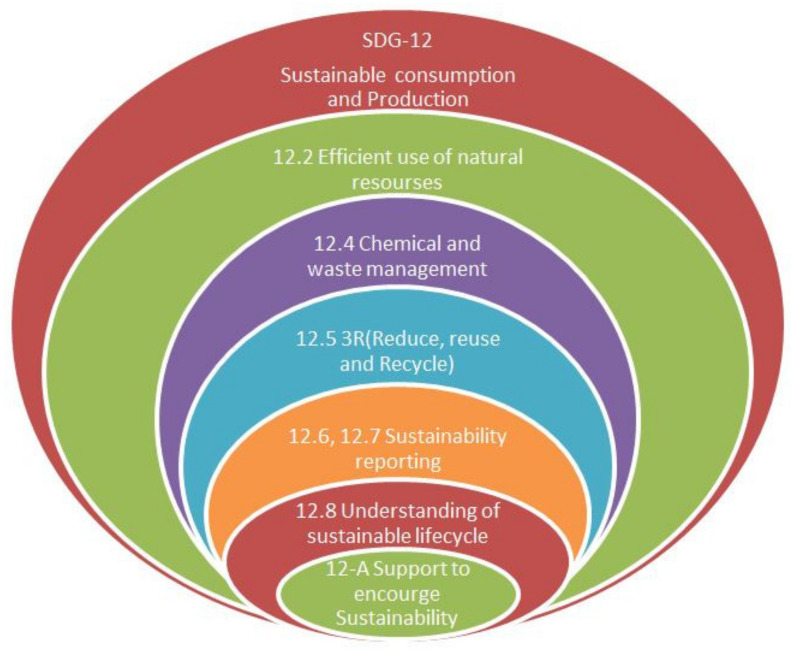
Key indicators of SDG-12.

**Figure 2:**
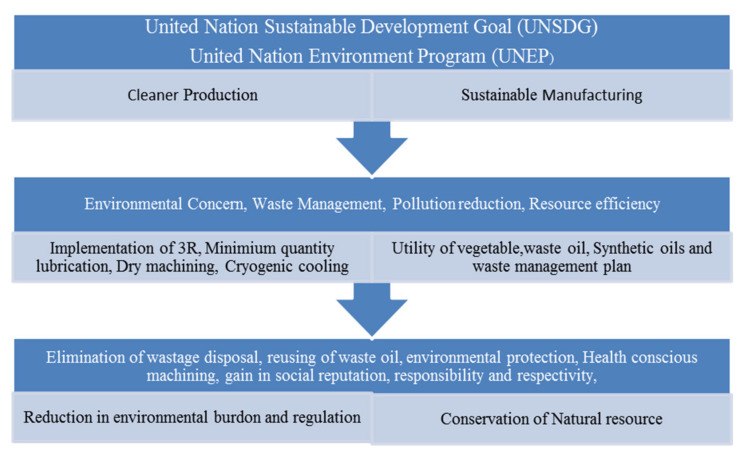
Targets of UNSDG and UNEP.

**Figure 3:**
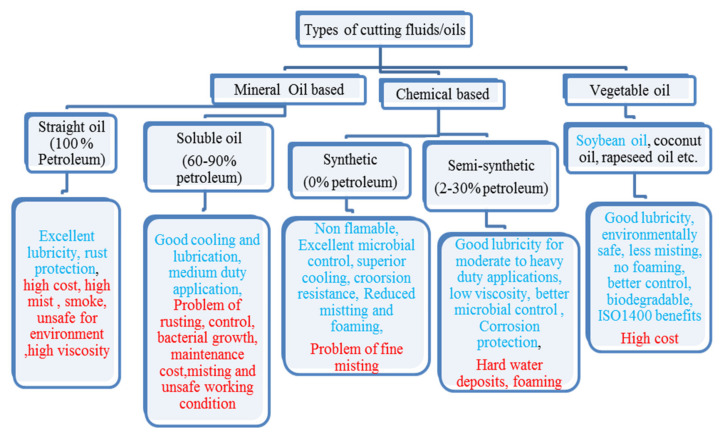
Description of cutting fluids merits and demerits [54,55].

**Figure 4:**
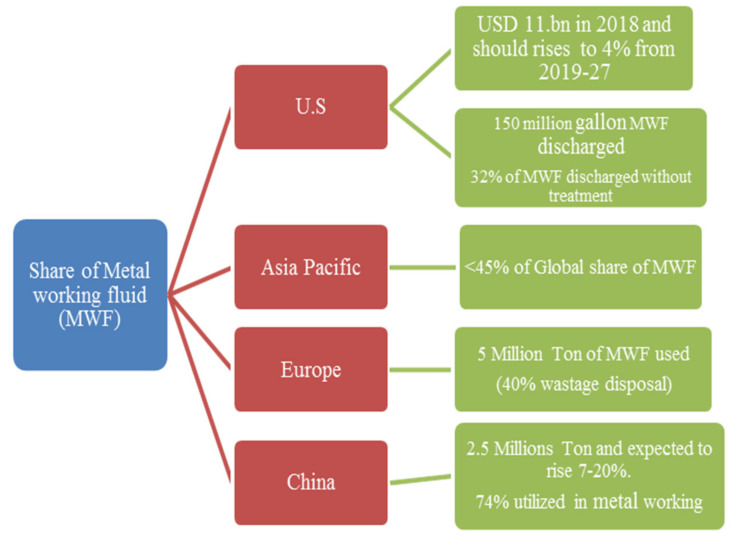
Overview of Global economy of MWF utilization [54,55,59,60].

**Figure 5:**
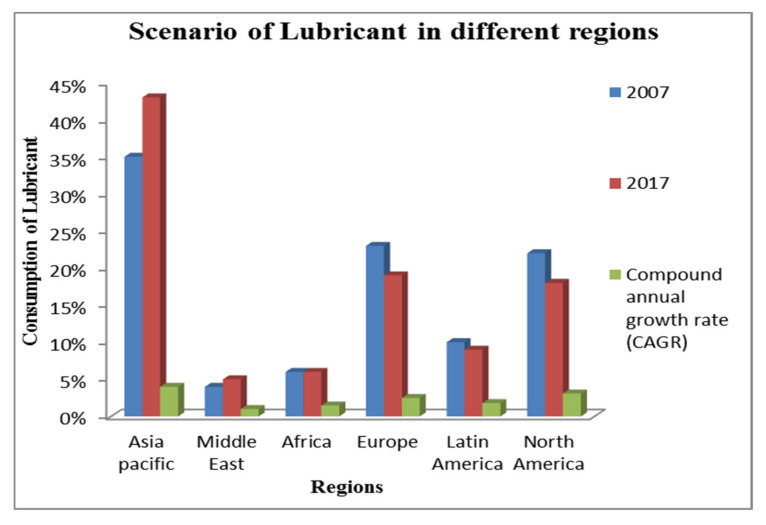
Analysis of lubricant consumption in different nations [59,60].

The consumption of natural resources is increasing in the present scenario due to a variable demand in different industrial sectors and can be fulfilled by utilizing cleaner energy such as solar, wind and hydropower. The details of SDG, UNEP, lubricant type and the economic analysis of petroleum-based cutting fluid, along with the global market share are mentioned in Figure 1, Figure 2, Figure 3, Figure 4 and Figure 5 that clarifies the need for reduction in resource consumption and wastage minimization to achieve the SDG. Therefore, waste minimization, reduction of non-renewable resources and environmentally friendly machining conditions are of great importance in the manufacturing sector. The UN sustainable development goal 12 (Figure 1) focuses on sustainable consumption and production. Presently, to fulfil the UNSDG, manufacturing organizations are facing strict environmental regulations regarding wastage reduction, carbon footprint, energy consumption, health hazards and resource efficiency. Hence, the utilization of sustainable cooling techniques, minimum quantity lubrication (MQL) and environmentally friendly oils (vegetable and synthetic oil) should be of major interest.

Further, to reduce the burden of wastage disposal, the utilization of waste motor oil along with MQL should be tested as a novel approach to machining super alloys. The exercise of all these inputs in manufacturing promotes a 3R approach that will not only reduce environmental hazards but also prove to be an economic advantage. Hence, to ensure sustainable manufacturing, the utilization of waste and vegetable oil (soybean) with MQL machining will minimize the gallons of cutting-fluid waste. Moreover, the comparative analysis of the machining performance of waste, vegetable and synthetic oils will ensure that the cleaner production has minimum costs associated with wastage disposal and recycling. Consequently, this will lead to a reduction in health hazards, pollution of water and soil that will conserve the aquatic life [53].

#### 1.1.2. Concept of Wastage Reduction and Its Impacts

In present age of the industrial scenario, organizations are focusing on the concept of green manufacturing for sustainable development to achieve the UN SDG. However, control of pollution associated with liquid wastage is a significant parameter which is still a major bottleneck in manufacturing industries utilizing cutting fluids. Therefore, wastage reduction plan is needed, especially for costs related to recycling, wastage disposal and environmental degradation.

Wastage minimization will not only impact environmental, but also economic performance through the execution of the UN sustainable development plan SDG-12. Similarly, the idea of cleaner production developed by the United Nations Environmental Program (UNEP) is also a great initiative to protect the environment. Hence, to accomplish the UNSDG-12 (Figure 2), along with UNEP’s aim in the manufacturing sector will not only eliminate the negative aspects of metal working fluids, but also provide economic consideration. Cutting fluid waste minimization has become the need of the hour among firms because of legal action from government agencies due to the poor waste management plans of cutting fluid disposal. Consequently, this influences environmental degradation in terms of soil, water and aquatic life. In addition to this, heavy fines may be imposed to the organizations disobeying the compliance of environmental protection. Thus, in such a scenario, the focus on fluid waste management plays a key role in addressing all issues that deal with cutting fluids [54]. The various properties of cutting fluids that should be monitored properly on the shop floor are corrosion protection, stability, transparency and viscosity along with health-related criterion like toxicity, flammability and misting. The selection of cutting fluids for particular machining operations is dependent upon a number of factors like the material being machined, parameter levels, cost, life expectancy, ease of fluid recycling, optimal concentration, storage practice and, most importantly, disposal cost. Basically, oil- and chemical-based cutting fluids are utilized these days for machining different categories of materials [55]. Moreover, the discharging of waste lubricants can affect the soil when it is dumped into sewers or leaked from oil containers. It may impact the physical, chemical and microbiological properties of the soil, and thus contaminate the groundwater, inhibiting the growth of living organisms due to an inappropriate supply of oxygen [56]. Sometimes, the heavy metals present in the oil prohibit the productivity of the soil. Further, wastage disposal can lead to the accumulation of various essential elements like P, K, Zn, Ca, Fe and Co together with non-required substances such as Al, Pb and Cd. Consequently, this can affect the tissues of plants [57,58]. When lubricant waste is illegally dumped into a water source, it adversely impacts the aquatic life due the formation of an oil layer on the water. Moreover, the continuous dumping may lead to fatal diseases like cancer and chronic fever. The combustion of waste oil is quite high and does not affect the environment as much, thus utilized as a raw fuel in boilers [53].

As represented in Figure 3, mineral oil has numerous demerits compared to chemical and vegetable-based oils and, hence, their application would restrict the attainment of the sustainable development goal. Therefore, the use of synthetic, vegetable and waste oils should be of much significance for cleaner production. Further, soybean oil has better machining performance due to the mentioned benefits and, therefore, has been utilized in the present investigations. Fungi-related problems are also associated with the maintenance of cutting fluids and, if it cannot be properly eliminated, leads to spoiling of fresh cutting oil that requires fungicide for its control [59].

#### 1.1.3. Global Scenario of Lubricant Market

The demand for lubricant has been increasing in recent years, despite COVID-19, as mentioned in Figure 4. Hence, to meet all these scenarios, the capacity has to be increased and will impact the degradation of natural resources. The interesting fact, as mentioned in Figure 4 and Figure 5, is that the demand for lubricant in the European and American regions has reduced because of the implementation of wastage management plans, strict government regulations and environmental consciousness. However, Asia Pacific is still reluctant to adopt a waste management plan along with the latest cooling/lubrication techniques, making it difficult to reduce the lubrication demand [54,55,59].

**Figure 6:**
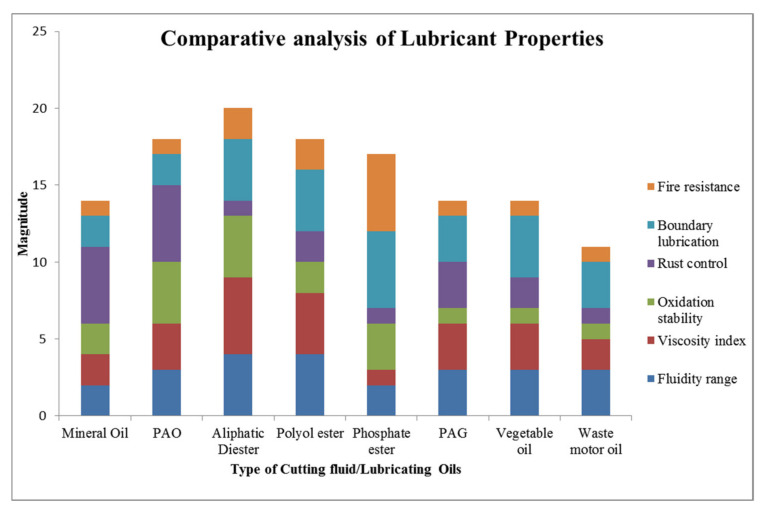
Comparative analysis of different lubricants [68].

**Figure 7:**
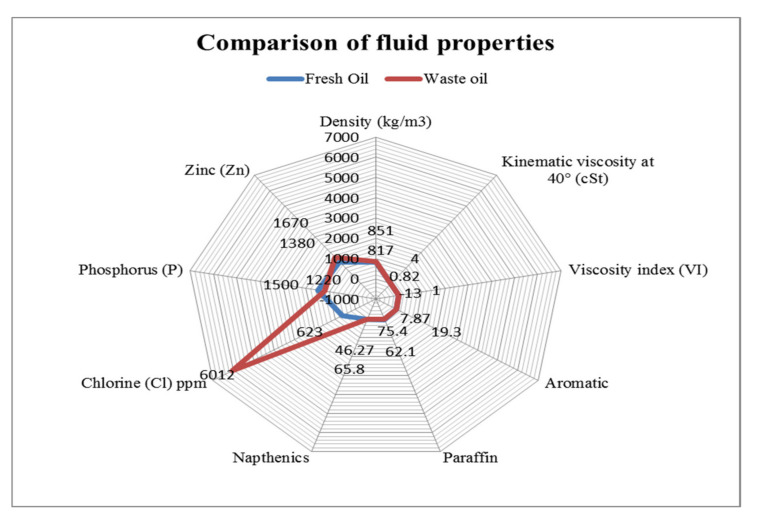
Comparative analysis of fresh and waste oil properties [69,70].

In the present scenario, different types of lubricant have entered into the market with less environmental impacts and enhanced resource efficiency. Thus, the usage of such types of oils should be a good alternative to the traditional mineral-based oil [61,62]. The demand for synthetic oil is increasing due to polyalphaolefins (PAO), synthetic esters and polyalkylene glycols (PAG) that have lower environmental hazards compared to mineral oil [44,45,63,64,65,66,67]. From Figure 6, it is clear that an aliphatic diester-based synthetic cutting fluid has the best performance in selected properties, followed by PAO and Polyol ester, then after that, phosphate ester. Although PAG and mineral oil have the same score, PAG has a better viscosity index, fluidity and boundary lubrication, but it trails in oxidation stability and rust control. Vegetable oil consists of glycerol and five types of fatty acids (Palmitic, Stearic acid, oleic, linoleic and linolenic acid in 10%, 4%, 18%, 55% and 13%, respectively) [45]; whereas waste oil contains the proportions of Cl, P, Zn, Napthalic and paraffin as chemical elements, mentioned in Figure 7. The flash point, boundary lubrication, viscosity index and fluidity of this oil are comparable with mineral oil.

Currently, environmental penalties and other regulations are forcing nations to reduce the consumption of cutting fluids, especially mineral oil. Therefore, in such situations synthetic and vegetable oils would be a good approach to protect the environment against wastage disposal and diminishing reserves of natural resources [54,60].

The properties of virgin oil are comparable [69,70], as the density of virgin oil is 817 kg/m^3^ and waste oil is 851 kg/m^3^. However, the kinematic viscosity is different to some extent. All other elements have a comparable range to waste oil. As mentioned in Figure 7, the density, viscosity and viscosity index (VI) of fresh and waste oils are not very distinguished; however, the chlorine content along with other elements are comparable. Petroleum-based cutting fluids are toxic when utilized and recycled continuously. In addition to this, it involves the cost of recycling equipment and wastage disposal too. Therefore, the selection of environmentally friendly synthetic and vegetable oils together with waste oil would be a good option to check their suitability as lubricants in machining operations. Waste oil is available at a price 5–8 times cheaper than fresh synthetic and vegetable oils. To mitigate the problems associated with petroleum-based cutting fluids, the environment protection agency (EPA) has recommended the use of environmental adaptable lubricant (EAL) formed from renewable energy resources, which are categorized as vegetable oil, synthetic oil and polyalkylene glycol. Further, aquatic toxicity is reduced while using these types of oils [59,60].

#### 1.1.4. Concept of 3R and Circular Economy

To fulfill the sustainable development goal, the concept of 3R along with idea of a circular economy would be the need of the present day industrial scenario to eliminate the wastage of cutting fluid and other oils [57].

**Figure 8:**
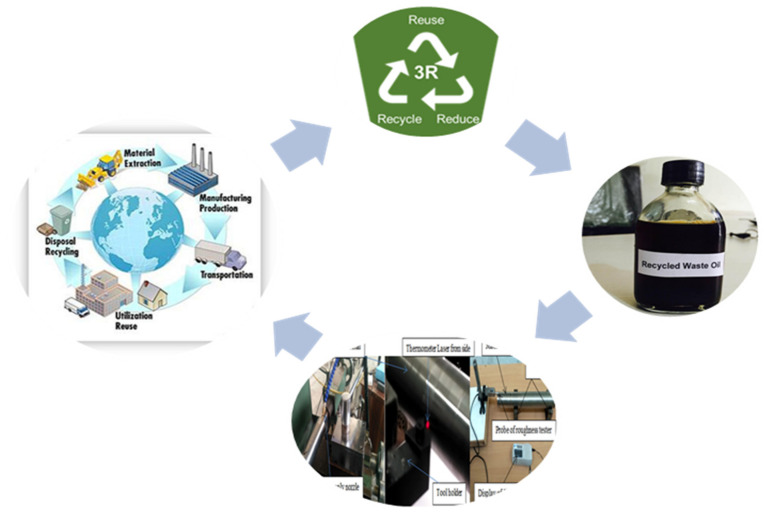
Concept of 3R and circular economy [1,71].

However, in the traditional approach, the raw material is produced, transformed into the required product and then waste is discharged into the atmosphere. However, the concept of a circular economy or 3R (Figure 8) pertains to reducing waste, recycling it and then utilizing it again to have zero waste at last. The studies [1,22,34] focused on the machining performance of Hastelloy under dry, flood and MQL cooling conditions, but are lacking with reference to the investigations of different categories of lubricants and their impact on surface roughness, temperature and chip reduction coefficient along with valid statistical analysis. Thus, in this work examination has been conducted on the turning of Hastelloy C-276 using different oils, such as synthetic, waste and soybean oils, so as to investigate their suitability as a lubricant in MQL-assisted machining. Moreover, the soybean oil (vegetable oil) has been selected due to its easy availability, environmentally friendly nature, use in past studies for machining different grades of steel, and rare application in machining of super alloys. In addition, the global warming potential (GWP) of vegetable-based oils such as soybean oil is less than 10, ensuring its viability as a suitable candidate for a lubricant oil to fulfil sustainability goals in the current era of sustainable manufacturing. The synthetic oil (ST KOOL) has been used because it provides minor environmental hazards and superior accomplishments compared to mineral based oils. Additionally, no research on the lubrication capability of waste oil has been conducted till date, and this study will give basic information for machining super alloy along with three different categories of environmental adoptable lubricants (EAL). The waste oil was collected from an automobile repair shop, where a special category of vehicles were repaired. The used oil (waste) is not further utilized for recycling and reusing, which would ultimately add disposal costs. In the present case, the waste motor oil has been filtered, and its physical and chemical properties have been tested prior to use. This is important because the utility of waste oil as a lubricant encourages the 3R approach of sustainable machining that eliminates wastage disposal costs, recycling requirements and minimizes the overall machining costs along with providing safer machining conditions and reducing the environmental burden. Therefore, an investigation on the different categories of environmentally compatible oils during MQL-based machining would provide a comprehensive study to enlist their lubrication capabilities for machining super alloys in view of all the negative aspects associated with the traditional as well as cryogenic cooling approach, because very limited investigation has been conducted on this material. Moreover, past studies have revealed that the machining of Hastelloy C-276 is tough and it needs effective cooling as well as a lubrication setup.

## 2. Experimental Planning and Methodology

Experimental Design, Workpiece, Tool and Lubricant Used

**Figure 9:**
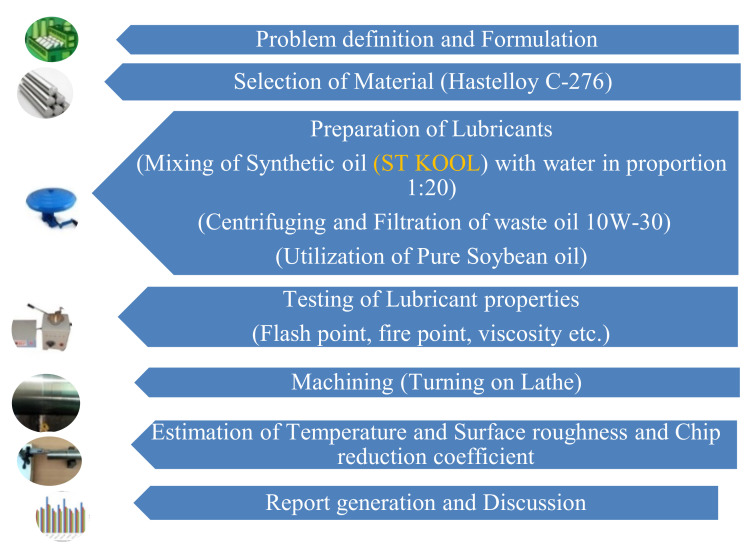
Work methodology.

An experimental investigation was conducted during turning of Hastelloy C-276 with work dimensions of 54 mm diameter and 550 mm length. The minimum quantity lubrication (MQL) was applied using different oils like synthetic oil (SO), Waste motor oil (WO) and vegetable oil (VO) at velocities of 53, 85 and 120 m/min, respectively. The step by step work methodology is illustrated in Figure 9.

**Figure 10:**
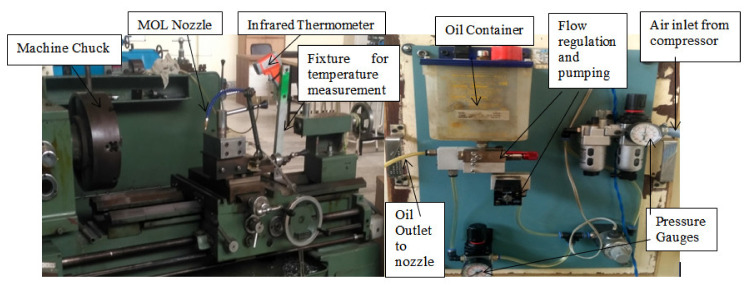
Snapshot of experimental setup for machining Hastelloy C-276.

**Figure 11:**
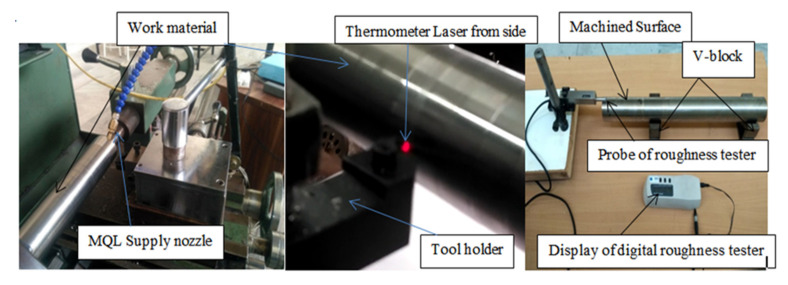
Picture of experimental setup.

Various properties of the workpiece and oils have been explored in Table 1 and Table 2. The output responses in the form of surface roughness (SR), chip reduction coefficient (CRC) and heat generation were evaluated at various ranges of input variables, keeping a constant nozzle gap, pressure and flow rate of the oil. The machining parametric ranges were selected based upon pilot experiments, expert opinion and firm guidelines. The tool-chip heat generation was recorded with a specially designed temperature-measuring setup, shown in Figure 10, which utilized a digital thermometer focused on the spot of higher temperature at a specified distance with a dual laser. Furthermore, the surface quality of the machined workpiece was evaluated with a HANDYSURF E-35B surface roughness tester as shown in Figure 11. On the other hand, the chip reduction coefficient was determined by the ratio of chip thickness after and before cutting using formula ζ (CRC) = t_c_/t. The thickness of the chip after cutting was measured with a digital micrometer as well as a vernier caliper. The thickness of the uncut chip was evaluated using relation t = f *Sin*K_r_, where f indicates feed rate and K_r_ is the side cutting edge angle (93°).

The design of the experiment was created on Design Expert Version-10, utilizing the central composite design (CCD) and having a total of 30 trials. A suitable number of pilot tests were conducted before experimentation and repetitions were performed to ensure the accuracy of outcomes. Further, the influences of input variables were examined through analysis of variance (ANOVA), and along with this, optimization was accomplished to enlist significant parameters.

The main properties of the different lubricants, such as flash point, specific gravity and viscosity, were analyzed before conducting of experiments to ensure better machining output and appropriateness for providing suitable lubrication action.

Synthetic oil (ST KOOL), soybean oil and waste oil were used as lubricants. The main properties of these three oils are mentioned in Table 2. There is no major difference in the properties of vegetable and synthetic oil. However, there is variation in flash point, kinematic viscosity, biodegradability and global warming potential (GWP). The lubrication assisted with the MQL system, along with low temperature and extreme pressure cooling, and has a considerable influence on lighter density alloys like titanium [36]. The details of the experimental setup, along with the parametric range, are mentioned in Table 3. The selection of a feed below 0.05 mm/rev has a great influence on the surface finish when the nose radius is less than unity and, therefore, it is an important variable which impacts the machining performance in finish turning [37].

The performance optimization of Hastelloy C-276 assisted with green-oil MQL in turning operations has been rarely available in the literature. Further, the utility of a circular economy (3R) is the need of the present-day scenario to minimize the environmental hazards and various costs associated with traditional mineral oil-based cutting fluids. Therefore, the performance optimization of the mentioned was carried out through the CCD approach of RSM to identify suitable ranges of machining parameters and their impact on output response. The experimental design and outcomes are listed in Table 4 and the same has been transformed for ANOVA and graphical analysis. In CCD, first order design consists of central and axial points that evaluate the 2nd order turning variables. The overall experimental design includes six center points, and a factorial point and axial point with alpha (α = 1). In the present experimentation, four input variables like cutting speed (v), feed rate (f), depth of cut (doc) and cooling conditions (C.C), along with three output responses such as SR, cutting temperature and CRC were analyzed in a total of 30 runs. The influence and optimization of input variables were investigated to find the suitable parameter ranges, having acceptable performance in all conditions. The flank wear was evaluated using a tool maker microscope for checking the lubrication action among all oils, and the same has been presented in the form of SEM micrographs

## 3. Results and Discussions

The output performance in terms of surface roughness, temperature and chip reduction coefficient are presented in Table 4. The optimization of input variables was performed through ANOVA utilizing Design Expert software, and the influence of all input variables are presented in the form of different graphs.

### 3.1. Analysis of Surface Roughness

Table 5 explores the ANOVA analysis of surface roughness with reference to various levels of input parameters and lists the important statistical terms of optimization. The Model F-value 81.518 in Table 5 specifies that the model is dominant, and there is only a 0.01% frequency that F-value this sizeable should occur due to error. Further, the *p*-value under 0.0500 signifies the validity of individual terms. In the present case, input factors like A, B, C, D, AD, A^2^, B^2^, C^2^, D^2^ have been found effective and the “Lack of Fit F-value” 2.86 implies that it is not valuable wrt the pure error. It manifests in only a 12.88% expectation of inaccuracy pertaining to error. The entity predicted R^2^ and adjusted R^2^ with a magnitude of 0.9494 and 0.9749 denoting the closeness of both terms, with each having reasonable agreement. For the accuracy of results, these values should be larger than 0.8. Further, as a thumb rule, the precision results should be more than 4 and in present case it is noted as 37.85. In addition, the results listed in Table 5 signify the accuracy of the experimental results with the predicted outcomes.

The percentage contribution to influencing surface roughness of input variables such as v, f, doc and CC were evaluated as 24.26%, 5.02% and 23% and 8.78%, respectively. This benefaction means that cutting speed is ascendant followed by depth of cut, cooling environment and feed rate. The final Equation (1) for surface roughness (SR), in terms of coded factors, is given by:

(1) $$\mathrm{SR}=0.61-0.18^{}\times \;A+0.082\times \;B+0.18\times \;C-0.011\times \;D-\;0.011\times \;\mathrm{AB}-0.0024\times \;\mathrm{AC}+0.054\times \;\mathrm{AD}-0.00375\times \;\mathrm{BC}-\;0.011\times \;\mathrm{BD}+0.00125\times \;\mathrm{CD}+0.18\times {\;A}^{2}+0.21\times {\;B}^{2}+0.070\times {\;C}^{2}-\;0.11\times {\;D}^{2}$$

#### 3.1.1. Statistical Plots for Surface Roughness

**Figure 12:**
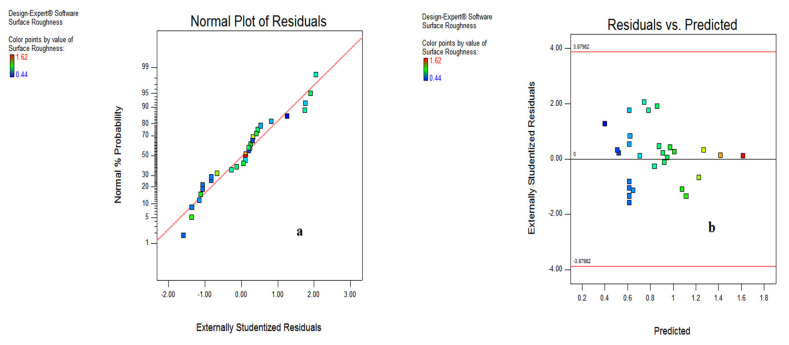
Normal plot of residual (**a**); residual vs. predicted (**b**).

**Figure 13:**
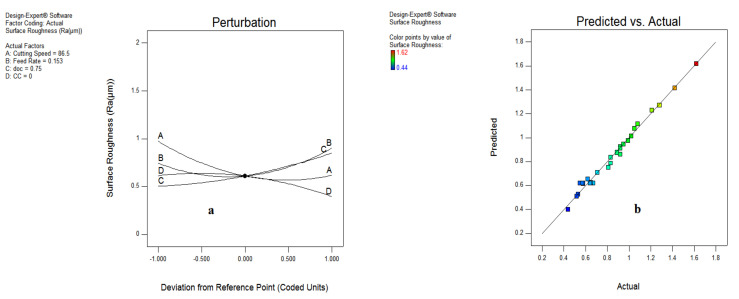
Surface roughness plot for perturbation (**a**); predicted vs. actual (**b**).

Figure 12 reveals the interaction effects amid various input variables on surface roughness together with predicted vs. actual outcomes. It indicates that the spread is almost close to the defined limit of predicted results, and the normal probability plot depicts that maximum observations are near to the line, which denotes the favorable experimental results. The perturbation chart shown in Figure 13a illustrates the influence of input variables starting from A’ and followed by B, D and C. However, on the right portion of the plot the parameters B, C, A and D have been listed. Further, Figure 13b shows that the spread of surface roughness adheres to the inclined line and suggest the mapping of the experimental results with the predicted values evaluated by the model.

#### 3.1.2. Impact of Input Variable on Surface Roughness

**Figure 14:**
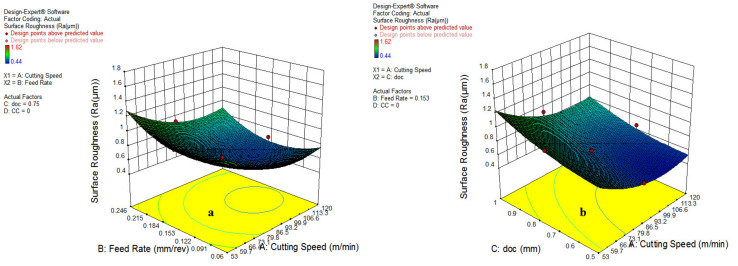
Influence of parameter on SR; cutting speed and feed rate (**a**); cutting speed and doc (**b**).

Figure 5 shows that the increment of cutting speed reduces the surface roughness. Though unlikely, the feed rate increases the surface roughness; while on the other side, the depth of the cut and the cooling environment increases surface roughness due to the impact of higher heat generation at the tool chip interface. As depicted in Figure 14a, it has been revealed that increasing the feed rate resulted in a surge of surface roughness because of the increase in chip load and area of cutting. Further, the higher speed enhances the surface finish due to the thermal softening at elevated temperatures and lower coefficient of friction, as visible in Figure 14b.

**Figure 15:**
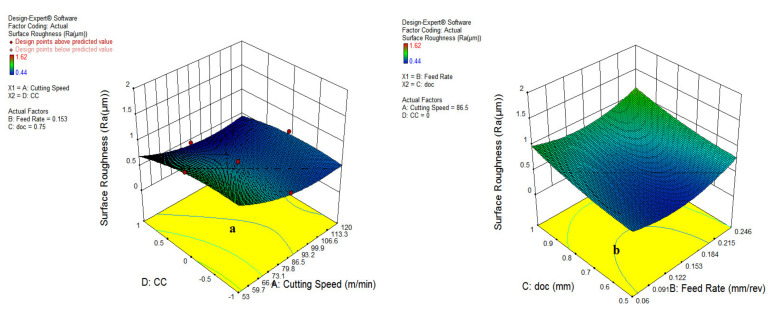
Impact of cutting speed and cooling type (**a**); feed rate and depth of cut (**b**).

The results shown in Figure 15b reveal that surface roughness is significantly dominated by the depth of cut and feed rate. However, a much lower impact of cooling type has been reported because of minor differences in cutting fluid properties, as represented in Figure 15a, which depicts that the vegetable and waste oil can also be used for machining operation.

#### 3.1.3. Contour Plot for Surface Roughness

**Figure 16:**
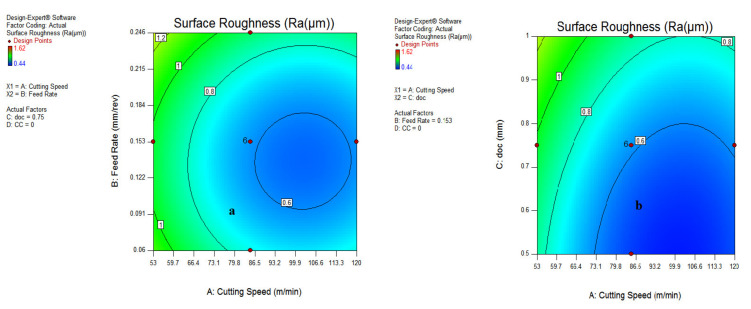
Contour plot for SR; cutting speed and feed rate (**a**); speed and doc (**b**).

**Figure 17:**
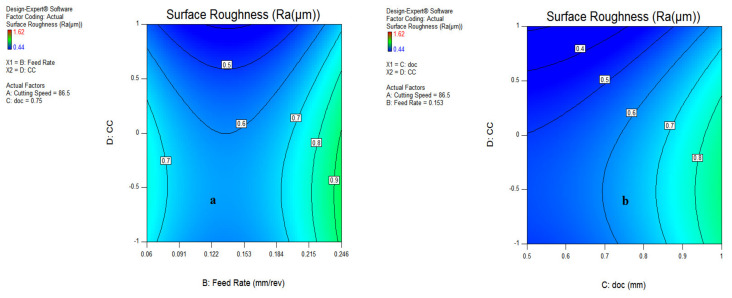
Contour plot for SR; feed rate and doc (**a**); doc and types of coolants (**b**).

The contour plots shown in Figure 16 and Figure 17 indicate the best region of surface roughness at different levels of input parameters. As shown in Figure 16, it has been revealed that the best surface finish was recorded at speeds of 80–118 m/min, having a feed rate of 0.11–0.16 mm/rev; while for the AC interaction plot in Figure 16b, the suitable range of surface roughness was observed at 73 m/min at a depth of cut of 0.75 mm. As visible in the different figures of contour plots, the darker blue region represents the good quality of surface finish at different ranges of process parameters. However, the greenish color signifies the surface roughness that requires improvement in machining conditions. Figure 17a depicts a favorable surface roughness during vegetable oil, coded as (0), and waste oil (1) because of their appreciable lubrication mechanism during machining operation. The region of good surface finish has been judged at a doc range of 0.5–0.85 mm, as illustrated in Figure 17b.

Similarly, an SR of 0.6 µm was found at a velocity of 86.5 m/min, a feed of 0.07 mm/rev along with a doc of 0.75 mm during vegetable and waste oil cooling conditions compared to the synthetic oil MQL system, as per the illustration in Figure 17a,b.

The interaction AC in Figure 16b illustrates that the SR of 0.6 µm was recorded at a speed range of 86–136 m/min, having depth of cut values from 0.5–0.7 mm. The good surface finish was found during vegetable oil MQL at 0.5–0.7 mm doc. From all contour plots, it has been revealed that the favorable SR was recorded during the vegetable and waste oil MQL at feed, speed and doc levels of 0.07–0.11 mm/rev, 86–136 m/min and 0.5–0.8 mm, respectively.

### 3.2. ANOVA Investigation for Cutting Temperature

The ANOVA analysis results mentioned in Table 6 illustrate that the Model F-value of 191.57 indicates it validity, as only a 0.01% chance of inaccuracy exists due to inappropriate signals. The model *p*-values less than 0.0500 for model terms A, B, C, AB, A^2^, B^2^, C^2^, and D^2^ signifies the crucial items in the model. Further, the tag Lack of Fit having an F-value of 1.85 implies non-dominance, which is always desirable, and there is a 25.79% expectation that it could happen because of bad signals. The percentage contribution of cutting speed, feed rate and doc was evaluated as 85.5%, 1.84% and 4.12%, respectively. Further, the percentage contribution of cooling type is significant due to the similarity in lubrication properties of cutting oil. However, it did reduce the temperature by a favorable amount. Furthermore, the close relation of predicted R^2^ and adjusted R^2^ signifies that the experimental results are within an acceptable range to the predicted outcomes. Moreover, the term adequate precision of 45.64 stipulates the suitable accuracy of the predicted and actual results for the cutting temperatures, having a standard deviation of 5.29 and mean value of 238.34. In addition, the square terms of A, B, C and D having a contribution of less than or equal to 1% signifies their minor impact.

The reason behind the non-significance of cooling type has been attributed to the identical ranges of temperature observed during the experimental results. In addition to this, the influences of input perimeters have been discussed with the help of 3D plots and contour chart. The final equation for temperature in terms of coded factors is as follows:

(2) $$\begin{array}{cc} \mathrm{Temp} & =258.778+59.722\times \;A+8.792\times \;B+13.11\times \;C-1.558\times \;D\\ & \;+3.0036\times \;\mathrm{AB}+1.3473\times \;\mathrm{AC}+0.599\times \;\mathrm{AD}\\ & \;+\left(-2.5\times \;\mathrm{BC}\right)+1.5\times \;\mathrm{BD}+1.625\times \;\mathrm{CD}\\ & \;+\left(-17.0153\right)\times {\;A}^{2}+\left(-17.807\right)\times {\;B}^{2}+\left(-7.807\right)\times {\;C}^{2}\\ & \;+10.193\times {\;D}^{2} \end{array}$$

#### 3.2.1. Statistical Plots for Cutting Temperature

**Figure 18:**
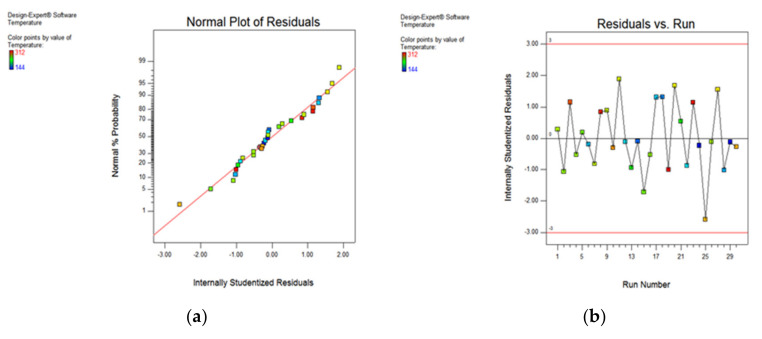
Plot for cutting temperature: normal probability (**a**) and residual vs. run (**b**).

The various types of statistical plots indicating the significance of output responses have been shown in the form of normal probability plot, residual versus run, predicted vs. actual and Box–Cox plots. These plots indicate that the responses are within the defined range and express the significance of the developed model. Figure 18a shows that the scatter of the cutting temperature values obtained during experimentation lies near to the normal probability line, while Figure 18b indicates that the residual vs. run entities of the cutting temperature are within the range of the −3 to 3.

As illustrated Figure 18b, it has been found that most of the values are within range (−2 to 2), and only a few values are out of the region (observation 25); otherwise, all other values are within the acceptable limit.

**Figure 19:**
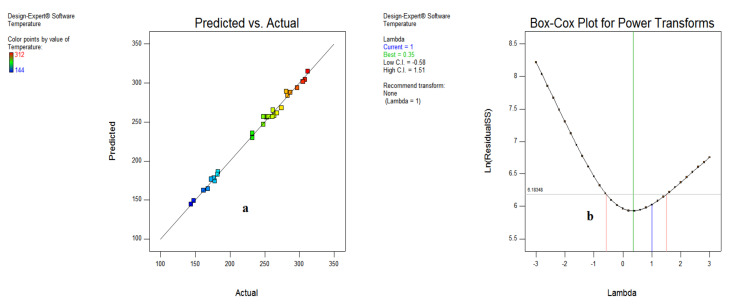
Predicted vs actual (**a**) and Box–Cox plot (**b**) for temperature.

As depicted in Figure 19a, the data points adhering to a straight line indicates a minor difference between actual and predicted results. Further, the Box–Cox plot in Figure 19b indicates 0.35 as the best value, along with the least (−0.58) and higher values (1.51), neglecting the transformation to normalize the output responses.

#### 3.2.2. Factor Affecting Cutting Temperature

**Figure 20:**
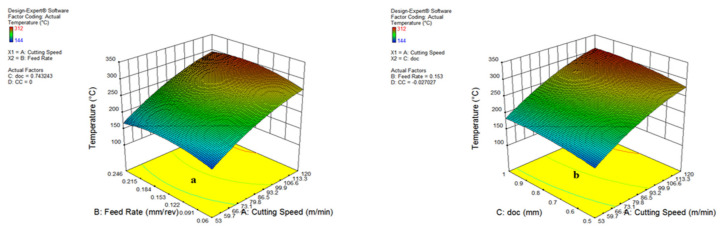
Impact of parameter on temperature: cutting speed and feed (**a**); cutting speed and doc (**b**).

**Figure 21:**
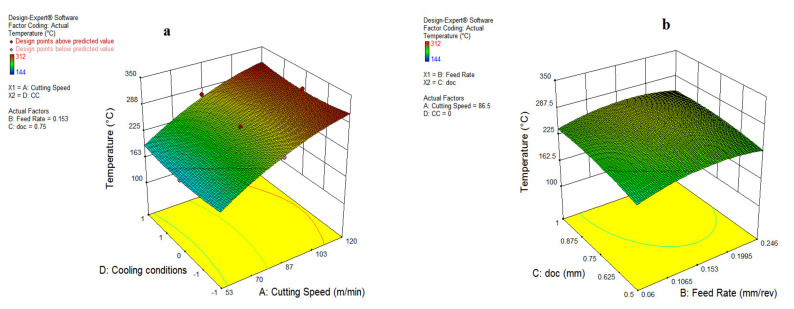
Impact of parameter on temperature: cutting speed and MQL type (**a**); feed and doc (**b**).

Figure 20 represents the impact of v and f on cutting temperature, which specifies that variations in the cutting speed and feed rate expand the cutting temperature. The surge of cutting temperature because of a rise in speed is due to the fact of higher friction and rubbing at various interactions of tool-work, which is expressed in Figure 20a,b. The higher heat formation occurred due to increments of cutting speed along with a rise in feed rate because of a larger cutting area and chip load [1]. In addition to this, a growth in the depth of cut also led to the cutting temperature shooting up due to an expansion of the cutting area ahead of the insert edge. The impact of different oils on heat generation is explored in Figure 21a.

The cooling environments have variations in temperature due to the cooling and lubrication action of different oils. The lowest temperature was found during vegetable oil, coded as (0), because of its significant lubrication ability and the cooling action of an air-assisted jet focused on the rake face of the cutting insert.

It is also noticeable from Figure 21a,b, that the waste oil (1) also reduced the temperature by a notable amount, owing to its good viscosity and handsome lubrication action. However, a few patterns of smoke were observed during the application of the waste oil, which needed to be modified by the addition of some additives, suitable exhaust and an efficient filtration system. As far as synthetic oil (−1) is concerned, an intermediate temperature was observed during experimentation. Furthermore, an improvement in the quality of synthetic oil would be quite useful to minimize the cutting temperature in future applications. From these experimental observations, it can be stated that the vegetable oil and waste oil have been found to be suitable candidates for minimizing cutting temperature.

#### 3.2.3. Contour Plot for Temperature

**Figure 22:**
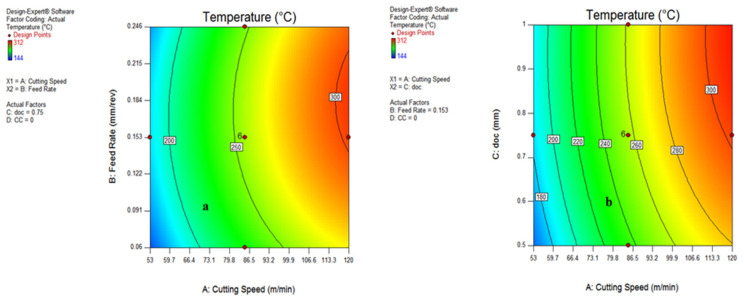
Contour plot for temperature: speed and feed (**a**); speed and doc (**b**).

**Figure 23:**
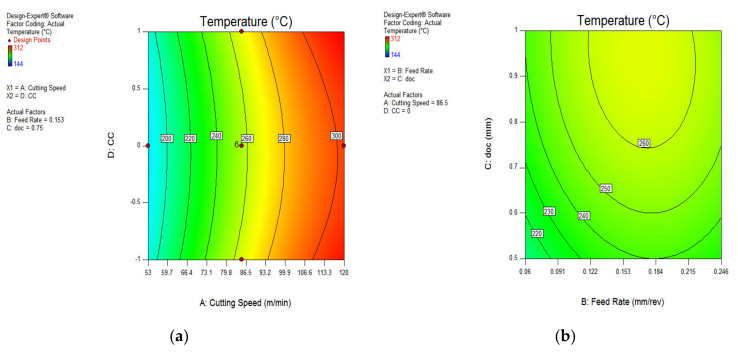
Contour plot for temperature: speed and MQL type (**a**); feed and doc (**b**).

The influence of various cutting parameters on temperature has been shown with the help of contour plots from Figure 22 and Figure 23. It signifies the range of input variables and output responses and can be used as a guide for setting the process parameters for the actual machining of difficult-to-machine materials.

Figure 22a,b depicts that the maximum temperature was determined at a speed range above 90 m/min, with a depth of cut of 0.75–1 mm.

The range of maximum cutting temperature for cooling conditions and feed rate have been evaluated in Figure 23a,b as vegetable oil (0) and 0.11–0.22 mm/rev, respectively. Further, it is noticeable that no major difference between the temperature range during different cooling conditions were reported. With the help of contour plots, the significant range of all input variables have been found and can be useful for further research.

### 3.3. ANOVA Examination for Chip Reduction Coefficient

The ANOVA table for CRC is represented in Table 7, comprising the details of input parameters and corresponding values of output responses accompanying various statistical terms. The F-value 31.53 stipulates that the model is valid and has less probability of inaccuracy as per the signals. The numerical *p*-value less than 0.05 for any item in the model expresses the validity, and for this case, A, B, C, D, BC, A^2^ and B^2^ are significant model terms. The percentage contribution of input parameters on CRC have been evaluated as feed rate (60%), doc (13.6%), cutting speed (9.2%) and coolant type (4.3%). Further, the Lack of Fit value of 1.90 implies the non-significance of invalid values with respect to pure errors.

The R^2^ and adjusted R^2^ values are more than 80%, representing the thrust of the developed model. In addition to this, the standard deviation and adequate precision is about 0.086 and 23.197, respectively, signifying the low magnitude of errors in the developed model. Finally, the predicted residual error sum square has been listed as 0.63, indicating the accuracy of the model for the evaluation of predicted outputs, with reference to each point in the model. The final equation for CRC in coded factors is given by Equation (3).

(3) $$\begin{array}{cc} \mathrm{CRC} & =1.95964+0.128722\times \;A+\left(-0.329503\right)\times \;B+0.156148\times \;C\\ & \;-0.087221\times \;D+\left(-0.00730\right)\times \;\mathrm{AB}+0.0347\times \;\mathrm{AC}\\ & \;+0.04402\times \;\mathrm{AD}+\left(-0.0621\times \;\mathrm{BC}\right)+0.0313\times \;\mathrm{BD}\\ & \;+\left(-0.0637\right)\times \;\mathrm{CD}+\left(-0.137245\right)\times {\;A}^{2}+0.265983\times {\;B}^{2}\\ & \;+0.0209925\times {\;C}^{2}+\left(-0.0117527\right)\times {\;D}^{2} \end{array}$$

#### 3.3.1. Statistical Plots for Chip Reduction Coefficient (CRC)

**Figure 24:**
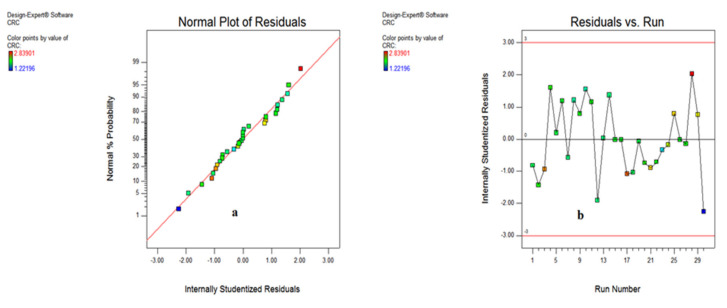
Normal probability (**a**) and residual vs. run plot (**b**) for CRC.

**Figure 25:**
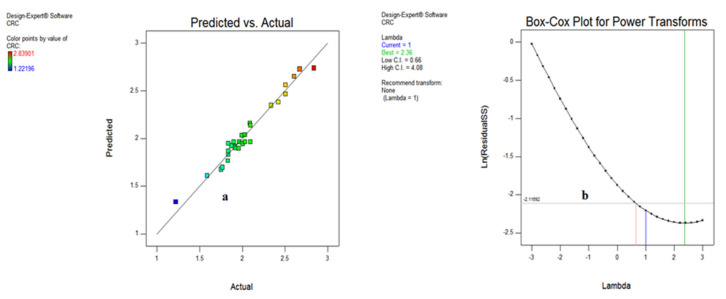
Predicted vs actual (**a**) and Box–Cox plot (**b**) for CRC.

The various plots indicating the significance of input variables on chip reduction coefficient are shown in Figure 24 and Figure 25 in the form of normal probability plot, residual versus run, predicted vs. actual and Box–Cox plots. The chart indicates that the responses are within the defined range and express the significance of the developed model. Figure 24a,b illustrates that the spread of CRC is clinging to the inclined straight line, ensuring the accuracy of the output data.

The residual vs. run data in Figure 24b specifies that the residual outcomes are lying between −2 to 2, except observation 30, signifying the accuracy of responses. In addition to this, the predicted vs. actual results illustrated in Figure 25a show that the whole entities of CRC are within close limit to the actual outcomes. Furthermore, the Box–Cox plot shown in Figure 25b indicates that there is no further transformation of data required for enhancing the accuracy of the developed model.

#### 3.3.2. Influence of Process Parameter on Chip Reduction Coefficient (CRC)

**Figure 26:**
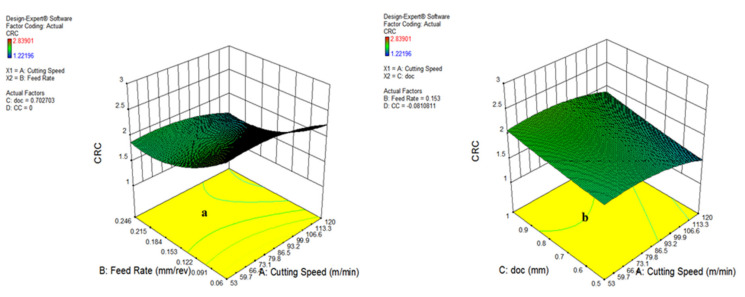
Impact of parameter on chip thickness: cutting speed and feed (**a**); cutting speed and doc (**b**).

**Figure 27:**
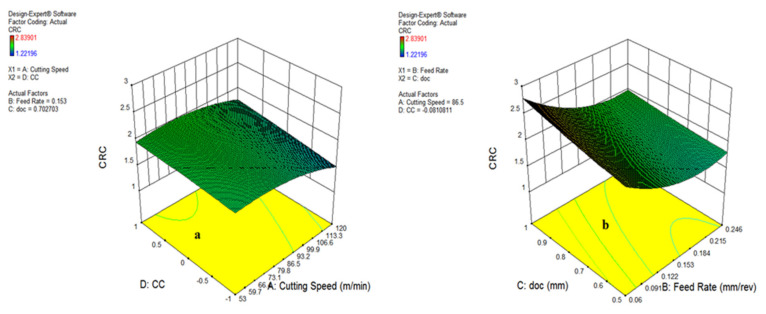
Impact of parameter on temperature: cutting speed and MQL type (**a**); feed and doc (**b**).

The Figure 26 and Figure 27 represent the impact of v, f, doc and cooling conditions (CC) on the chip reduction coefficient. It has been noticed from the plots that, on varying the aforementioned variables, the CRC changes. On increasing the cutting speed, the CRC initially increases and then reduces, as visible in Figure 26a. However, the increments of doc reversed the impact on the chip reduction coefficient depicted in Figure 26b. In addition to this, the minor impact of cooling conditions was noticed on CRC, which is illustrated in Figure 27a, because of a similarity of cutting fluid properties. Figure 27b signifies that a rise in doc and feed rate reduces the CRC, as mentioned earlier, due to the impact of change in the cutting area ahead of tool and variation in chip thickness at defined input variables. In addition, the lowest CRC was observed during vegetable oil compared to the other oils.

#### 3.3.3. Contour Plot for Chip Reduction Coefficient (CRC)

**Figure 28:**
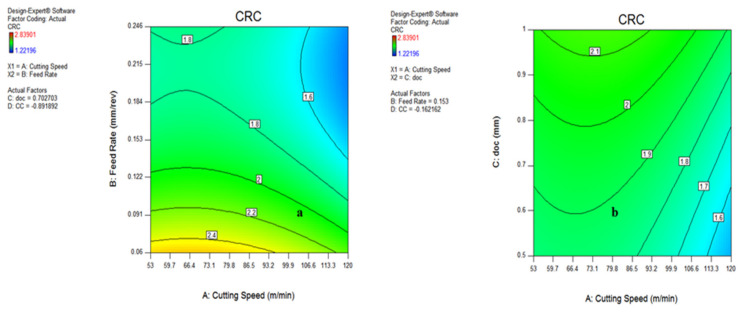
Contour plot for CRC: speed and feed (**a**); speed and doc (**b**).

**Figure 29:**
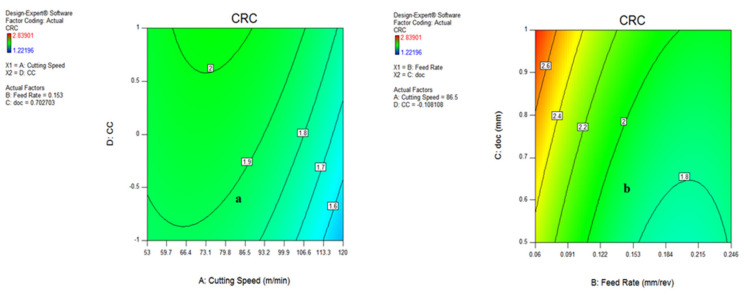
Contour plot for chip thickness: speed and MQL type (**a**); feed and doc (**b**).

The influence of various process parameters on CRC have been shown with the help of contour plots that illustrate the range of input variables and output response for defining the process parameters during actual machining. Figure 28a indicates that the chip reduction coefficient reduces as the v and f are expanded, because of a change in the uncut chip thickness. However, with the involvement of the depth of cut, the CRC reduces up to a certain limit and then starts increasing with the increase in v and doc, both due to the effect of more load on the shear area and a deformation zone, represented in Figure 28b. There is a sharp increase in CRC beyond a cutting speed of 86.5 m/min because of increments in cutting forces in the shear zone, which is directly indicated by the chip reduction coefficient. The variation of CRC wrt speed vs. cooling conditions, as well as feed rate vs. depth of cut, have been shown in Figure 29a,b, indicating that there is a minor variation in CRC in different cooling conditions. However, the CRC surges with respect to cutting speed, along with various cooling conditions as well. On the other side, CRC rises sharply with a depth of cut up to 0.122 mm/rev, and then reduces because of a change in the uncut chip thickness, as represented in Figure 29b. The higher value of CRC was noticed at a lower value of feed rate, as illustrated in Figure 28 and Figure 29.

### 3.4. Evaluation of Desirability

During optimization of a multiresponse, it is required that the best levels of input parameters are achieved to augment the performance of the system. In analysis, the objective is to achieve maximum desirability for all output responses, having a smaller-the-better approach for surface roughness, cutting temperature and CRC.

**Figure 30:**
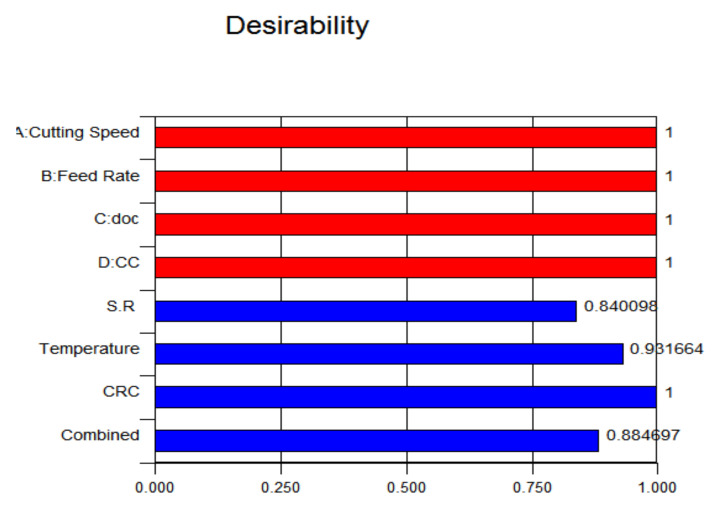
Desirability plot.

The optimization results, as per the responses, have been calculated using ANOVA analysis. The individual optimal desirability of the input parameters was set at 1 shown in red colour and further the optimal response desirability of each output parameter were evaluated by said approach illustrated in blue colour. It has been found in Figure 30 that maximum desirability of output response is achieved for CRC (1), followed by temperature (0.93) and surface roughness (0.84) that are almost near to or more than 0.80. Further, the combined desirability of the system is 0.88, which is also greater than 0.8, indicating that the set values of the input parameters are within the range of acceptable levels (0.80).

### 3.5. Confirmation of Model

**Figure 31:**
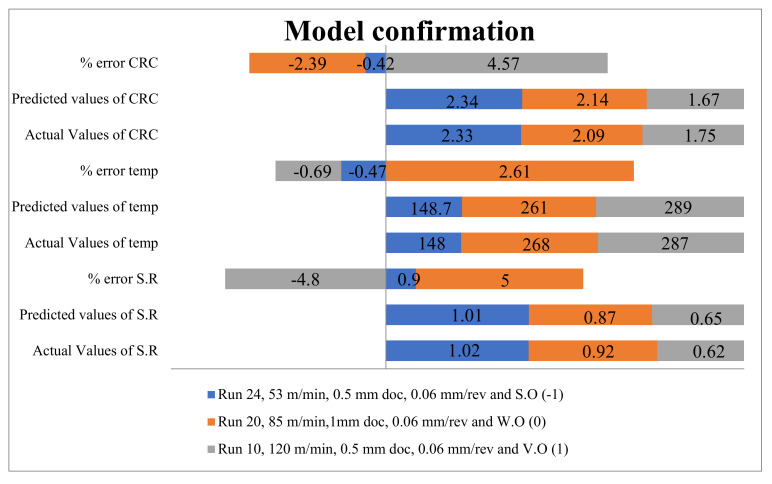
Model validation and performance comparison.

The model validation was tested in terms of percentage of error, actual and predicted results for all three output responses. Figure 31 indicates the actual and predicted values for CRC, temperature and SR in all three confirmation runs. The results for (CRC and temp) are very close to each other, whereas the maximum percentage of error for SR has been evaluated as 5%, in the case of waste-oil run 24.

**Figure 32:**
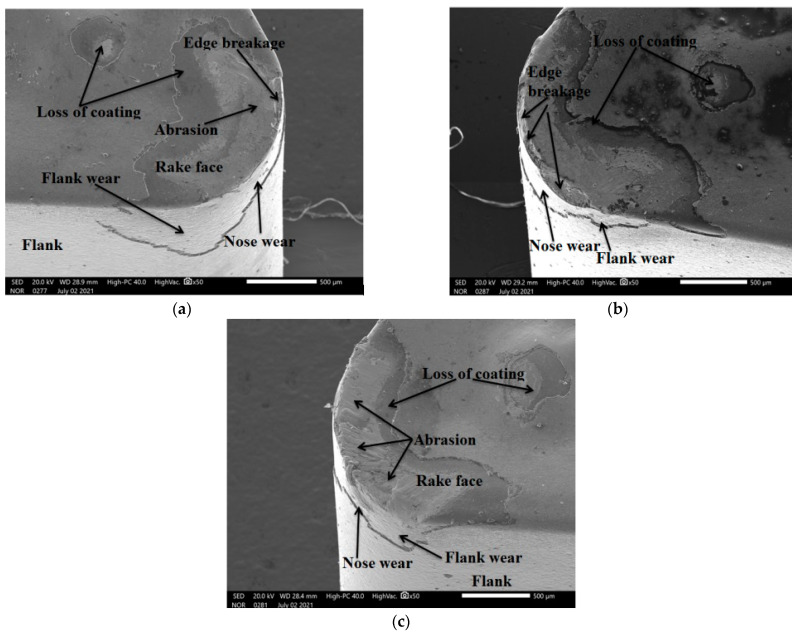
SEM image of tool wear in S.O (**a**), V.O (**b**) and W.O (**c**).

**Figure 33:**
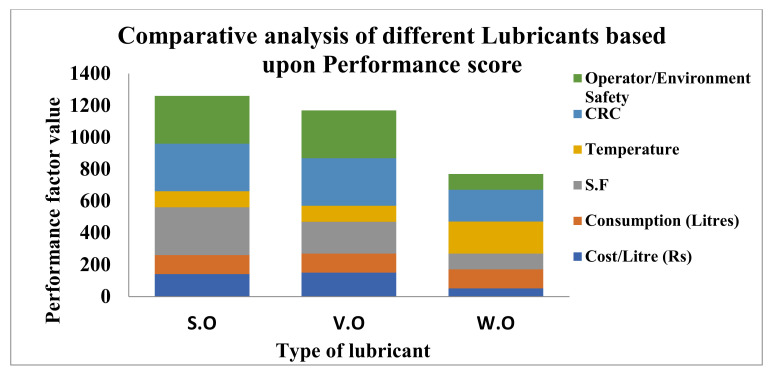
Analysis of lubricant performance.

In addition to this, the maximum % of error in temperature and CRC has been evaluated as 2.61 and 4.57%, respectively. It is noticeable that waste oil has a maximum percentage error in SR and temperature, while the least error in CRC. From Figure 31, Figure 32 and Figure 33, it is clear that the lowest temperature was recorded in synthetic oil, whereas the good surface finish and low CRC were recorded in vegetable oil. The waste oil had intermediate performance in all three output responses. Therefore, it can be concluded that waste oil performance is comparable with other oils to minimize the disposal cost and utility of consumption lubrication, validating the circular economy.

### 3.6. Analysis of Tool Wear

For the analysis of lubrication action during different oils, the tool wear has been evaluated, keeping a constant machining time of 90 s for each nine conditions. To minimize the machining cost, time and workpiece availability, only nine experiments were conducted for the evaluation of tool wear. The experimental results depicted in Table 8 revealed that flank wear increases as the cutting speed and feed rate are varied due to increments in friction and temperature. The SEM micrograph of flank wear shown in Figure 32 indicates that a loss of coating, nose wear and flank wear were reported during all lubrication conditions.

Abrasion wear was reported during waste oil (Figure 32c) due to the lower lubrication action at higher cutting speeds, leading to higher friction because of the rubbing action between the chip and tool; whereas a loss of coating and edge breakage along with nose and flank wear have been reported in the case of vegetable oil, as shown in Figure 32b. In addition to this, the same wear behavior exists for synthetic oil, as shown in Figure 32a. As per tool life criterion ISO-3685, the tool life ends when flank wear is 0.3 mm and, accordingly, failure occurred at speeds of 120 m/min in S.O as well as V.O, while for W.O the life ended at 85 and 120 m/min. All these observations indicate that higher tool wear occurs during the turning of Hastelloy C-276.

## 4. Cost Benefits Analysis of MQL with Other Techniques

Figure 33 represents a comparative analysis of performance among different lubricants with reference to cost, consumption and environmental considerations. The maximum score (3) has been granted to excellent performance, and a low score (1) for poor conditions. It is clear from the plot (Figure 33) that the highest score was secured by the synthetic oil (S.O), followed by vegetable oil (V.O) and, afterward, waste oil (W.O). The cost of vegetable and synthetic oils are not much different, while the cost of waste oil is 0.30 times the cost of others oils. However, filtering and other processing costs are associated with waste oil. The different colors represent the various performance terms and other factors that have a significant impact on economic, ecological and health aspects. The maximum cost was found in V.O and S.O, whereas the maximum surface finish was recorded in synthetic oil.

As far as operator safety is concerned, the vegetable oil is the most suitable. The lowest cost was found in waste oil, along with comparable CRC and acceptable levels of surface roughness. The utility of wastage oil reduces the disposal cost and, ultimately, minimizes the negative impacts on the environment in terms of aquatic life, soil and water pollution during its safe discharge. Hence, reusing waste oil is a good initiative for enhancing the machining performance of super alloy and other materials. As per a market survey and literature review, the cost-benefit analysis is illustrated in Table 9, indicating various factors for sustainable cooling techniques.

For the comparative analysis, 6 days single-shift, 6 h and 52 weeks were considered. The flow rate of different coolant/lubricants were taken from literature data as well as according to the present investigation. In Table 9, MQL with vegetable, synthetic and waste oils is cheaper than other types of cooling/lubrication techniques based upon cost, performance and environmental aspects. However, cryogenic cooling as well nano-fluid MQL machining have been found costly because of a larger consumption of coolant and the requirement of a suitable setup; whereas the conventional flood lubrication, comprising the expense of wastage disposal and health hazards, restrain its application. Hence, the utilization of green lubricants such as vegetable, synthetic and refined-used oils are good alternatives to other strategies for machining difficult-to-cut materials.

## 5. Conclusions

On the basis of the experimental results, and optimization analysis, the undermentioned conclusions have been extracted.

- Machining of Hastelloy C-276 is hard; therefore, the application of an efficient cooling and lubrication system is required to enhance the surface quality, diminish heat generation and minimize CRC at various ranges of input variables.
- Cutting velocity and feed rate has an inverse impact on surface roughness; further, the variation in depth of cut and coolant type alter surface roughness due to the impact of higher heat generation and differential cooling action.
- As per the experimental observations, surface roughness is highly influenced by depth of cut as well as feed rate; however, cutting oils have no major difference for surface finish, which indicates that vegetable oil and waste oil can also be applied for metal machining.
- The SEM micrographs reported a loss of coating, nose and flank wear during all lubrication conditions. Further, tool failure occurred at speed of 120 m/min in S.O as well as V.O, while for W.O the life ended at 85 and 120 m/min.
- The implementation of environmental adaptable lubricants, MQL and circular economy techniques is an approach to achieve the UN sustainable goal (SDG-12). Its overall impact on machining performance, along with the environment, has been listed in Table 10.
- The least temperature was found during the vegetable oil because of its significant lubrication action along with air-assisted jet aimed at the rake face of the cutting insert. Further, tt was been noticed that the waste oil reduced the temperature by a notable amount; despite its good lubrication action. However, a few patterns of smoke were observed during the application of the waste oil.
- Chip reduction coefficient is majorly impacted by cutting speed and feed rate, but not influenced by coolant type due the similar cooling and lubrication obtained in different conditions. However, a lower CRC was observed during vegetable oil cooling compared to other oils.
- With a rise in v and f, the chip reduction coefficient reduces because of a change in uncut chip thickness. However, with the involvement of depth of cut, the CRC reduces up to a certain limit and then start increasing.
- As per ANOVA analysis, the cutting speed majorly influenced the SR, significantly trailed by depth of cut, coolant type and feed rate. Subsequently, identical trends were also recorded for the first two variables, but a non-significance of coolant types was noted during cutting temperature. However, CRC was dominated by feed rate accompanied with depth of cut, cutting speed and coolant type.
- The maximum percentage of error for SR, temperature and CRC was found as 5, 2.61 and 4.57%, respectively, which means that the models are significant.
- The combined desirability of the system is 0.88, which is greater than 0.8, indicating that the set values of the input parameters are within the range of acceptable levels.

## Figures and Tables

**Table 1:** Chemical composition and mechanical properties of Hastelloy C-276 [1].

Element	C	Si	Mn	P	S	Ni	Cr	Mo	V	W	Co	Fe
Wt%	0.08	0.03	0.4	0.01	0.01	57.9	15.4	16	0.05	3.7	0.3	5.5
**Hardness**		**Elongation (%)**			**Tensile Strength**				**Yield Strength**			
84 HV		68			106 (ksi)				47.9 (ksi)			

**Table 2:** Important properties of lubricants.

Properties	Synthetic Oil (ST KOOL)	Vegetable Oil (Soybean Oil)	Waste Motor Oil (10W-30)
Flash point	230 °C	240 °C	120 °C
Specific gravity	0.88	0.91	0.93
Kinematic viscosity @ 40 °C (cSt)	32	33	56
Biodegradability [71,72]	20–30%	95%	10–15%
GWP kg of CO_2_ [73]	43–48	3	54

**Table 3:** Details of experiment elements.

S.N	Element	Details
1	Machine utilized	Centre Lathe (Poland made AFM TUG-40) 5.5 kW
2	Material used	Hastelloy C-276, (Φ 0.054 m × 0.55 m length)
3	Cutting tool insert	TNMG160408
4	Tool holder	MTJNR2525M16
5	Tool angles	−7°, −7°, 7°, 93°, 93°, 0.8 mm
6	Machining velocity (m/min)	53, 85 and 120 (m/min)
7	Coolants	Synthetic Oil, Vegetable oil and waste motor oil (−1, 0, +1)
8	Feed rate	0.06, 0.153 and 0.246 mm/rev (−1, 0, +1)
9	Depth of cut (doc)	0.5, 0.75 and 1 mm
10	MQL details	Air pressure 5 bar, flow rate of 90 mL/h
11	Nozzle distance (mm)	35 mm at 45° targeted at rake face of insert
12	Compressor	Ingersoll Rand
13	Roughness tester	HANDYSURF E-35B, TOKYO SEIMITSU
14	Heat measurement (°C)	Digital infrared thermometer MTQ580

**Table 4:** Experimental design and outcomes.

Standard	Run	Cutting Speed (m/min)	Feed Rate (mm/rev)	doc (mm)	C.C	SR (Ra µm)	Temp (°C)	CRC
1	24	53	0.06	0.5	−1 (S.O)	1.02	148	2.33
2	11	120	0.06	0.5	−1 (S.O)	0.58	264	2.00
3	14	53	0.246	0.5	−1 (S.O)	1.21	162	1.83
4	30	120	0.246	0.5	−1 (S.O)	0.83	283	1.22
5	17	53	0.06	1	−1 (S.O)	1.42	178	2.67
6	25	120	0.06	1	−1 (S.O)	0.92	281	2.50
7	6	53	0.246	1	−1 (S.O)	1.62	177	1.95
8	23	120	0.246	1	−1 (S.O)	1.05	308	1.58
9	29	53	0.06	0.5	1 (W.O)	0.71	144	2.42
10	2	120	0.06	0.5	1 (W.O)	0.53	253	2.08
11	18	53	0.246	0.5	1 (W.O)	0.89	168	1.87
12	10	120	0.246	0.5	1 (W.O)	0.62	287	1.75
13	28	53	0.06	1	1 (W.O)	1.08	173	2.83
14	3	120	0.06	1	1(W.O)	0.83	297	2.60
15	22	85	0.246	1	1 (W.O)	1.28	183	1.99
16	19	85	0.246	1	1(W.O)	0.95	312	1.91
17	12	85	0.153	0.75	0 (V.O)	0.99	182	1.83
18	8	85	0.153	0.75	0 (V.O)	0.67	305	1.76
19	21	85	0.06	0.75	0 (V.O)	0.81	232	2.50
20	5	85	0.246	0.75	0 (V.O)	0.92	248	1.91
21	13	85	0.153	0.5	0 (V.O)	0.52	232	1.83
22	20	85	0.153	1	0 (V.O)	0.92	268	2.09
23	7	85	0.153	0.75	−1 (S.O)	0.65	262	1.83
24	27	85	0.153	0.75	1 (W.O)	0.44	274	2.03
25	15	85	0.153	0.75	0 (V.O)	0.64	248	1.96
26	1	85	0.153	0.75	0 (V.O)	0.58	258	1.89
27	9	53	0.153	0.75	0 (V.O)	0.55	261	2.03
28	16	120	0.153	0.75	0 (V.O)	0.58	254	1.96
29	4	53	0.153	0.75	0 (V.O)	0.57	254	2.09
30	26	120	0.153	0.75	0 (V.O)	0.57	256	1.96

**Table 5:** ANOVA table for surface roughness.

Analysis of Variance Table for Surface Roughness						
Source	Sum of Squares	df	Mean Square	F-Value	*p*-Value Prob > F	
Model	2.39474	14	0.17105	81.51888	0.00000	significant
A-Cutting Speed	0.58320	1	0.58320	277.93616	0.00000	
B-Feed rate	0.12005	1	0.12005	57.21234	0.00000	
C-doc	0.55476	1	0.55476	264.38036	0.00000	
D-Cooling type	0.21561	1	0.21561	102.75134	0.00000	
AB	0.00197	1	0.00203	0.96506	0.34150	
AC	0.00912	1	0.00902	4.30105	0.05572	
AD	0.04623	1	0.04623	22.02949	0.00029	
BC	0.00023	1	0.00023	0.10723	0.74785	
BD	0.00203	1	0.00203	0.96506	0.34150	
CD	0.00002	1	0.00002	0.01191	0.91453	
A^2^	0.092	1	0.08386	39.96698	0.00001	
B^2^	0.12	1	0.11967	57.02984	0.00000	
C^2^	0.013	1	0.01266	6.03513	0.02669	
D^2^	0.029	1	0.02861	13.63589	0.00217	
Residual	0.03147	15	0.00210			
Lack of Fit	0.027	10	0.00268	2.86030	0.12881	not significant
Pure Error	0.00468	5	0.00094			
Cor Total	2.42622	29				
Std. Dev.		0.046		R^2^		0.9870
Mean		0.83		Adj R^2^		0.9749
C.V. %		5.51		Pred R^2^		0.9494
PRESS		0.12		Adeq Precision		37.585

**Table 6:** ANOVA table for cutting temperature.

Analysis of Variance Table for Cutting Temperature						
Source	Sum of Squares	df	Mean Square	F-Value	*p*-Value Prob > F	
Model	75,053.6	14	5360.97	191.57	<0.0001	Significant
A-Cutting speed	64,201.39	1	64,201.39	2294.19	<0.0001	
B-Feed rate	1386.89	1	1386.89	49.56	<0.0001	
C-doc	3094.22	1	3094.22	110.57	<0.0001	
D-Cooling type	43.56	1	43.56	1.56	0.2313	
AB	144	1	144	5.15	0.0385	
AC	30.25	1	30.25	1.08	0.315	
AD	6.25	1	6.25	0.22	0.6433	
BC	100	1	100	3.57	0.0782	
BD	36	1	36	1.29	0.2745	
CD	42.25	1	42.25	1.51	0.2381	
A^2^	530.34	1	530.34	18.95	0.0006	
B^2^	821.55	1	821.55	29.36	<0.0001	
C^2^	157.91	1	157.91	5.64	0.0313	
D^2^	269.19	1	269.19	9.62	0.0073	
Residual	419.76	15	27.98			
Lack of Fit	330.43	10	33.04	1.85	0.2579	not significant
Pure error	89.33	5	17.87			
Cor total	75,473.37	29				
Std. dev.	5.29			R^2^		0.9944
Mean	238.23			Adj R^2^		0.9892
C.V. %	2.22			Pred R^2^		0.9703
PRESS	2240.06			Adeq Precision		45.640

**Table 7:** ANOVA table for chip reduction coefficient.

Analysis of Variance Table for CRC						
Source	Sum of Squares	df	Mean Square	F-Value	*p*-Value Prob > F	
Model	3.23	14	0.23	31.53	<0.0001	significant
A-Cutting Speed	0.30	1	0.30	40.75	<0.0001	
B-Feed rate	1.95	1	1.95	267.03	<0.0001	
C-doc	0.44	1	0.44	59.97	<0.0001	
D-MQL type	0.14	1	0.14	18.71	0.0006	
AB	8.546 × 10^−4^	1	8.546 × 10^−4^	0.12	0.7373	
AC	0.019	1	0.019	2.64	0.1250	
AD	0.031	1	0.031	4.24	0.0573	
BC	0.062	1	0.062	8.45	0.0108	
BD	0.016	1	0.016	2.14	0.1637	
CD	6.501 × 10^−4^	1	6.501 × 10^−4^	0.089	0.7698	
A^2^	0.049	1	0.049	6.64	0.0210	
B^2^	0.18	1	0.18	25.05	0.0002	
C^2^	1.142 × 10^−3^	1	1.142 × 10^−3^	0.16	0.6984	
D^2^	3.579 × 10^−4^	1	3.579 × 10^−4^	0.049	0.8280	
Residual	0.11	15	7.318 × 10^−3^			
Lack of Fit	0.087	10	8.690 × 10^−3^	1.90	0.2482	not significant
Pure Error	0.023	5	4.575 × 10^−3^			
Cor Total	3.34	29				
Std. Dev.	0.086		R^2^		0.9671	
Mean	2.04		Adj R^2^		0.9365	
C.V. %	4.18		Pred R^2^		0.8115	
PRESS	0.63		Adeq Precision		23.197	

**Table 8:** Experimental results.

S.N	Cutting Speed (m/min)	Feed Rate (mm/rev)	doc (mm)	Cutting Fluids/ Lubricant	Flank Wear VB (µm)
1	53	0.06	1	(S.O)	195.6
2	85	0.153	0.75	(S.O)	254.9
3	120	0.06	1	(S.O)	367.7
4	53	0.153	0.75	(V.O)	121.4
5	85	0.153	0.75	(V.O)	180.8
6	120	0.153	0.75	(V.O)	332.4
7	53	0.06	1	(U.O)	212.8
8	85	0.153	0.75	(U.O)	417.1
9	120	0.06	1	(U.O)	510.9

**Table 9:** Cost-benefit analysis among cooling strategies.

S.N	Lubricant/ Coolant	Cooling Methodology	Cost/L (₹ Rs)	Consumption (L/year)	Yearly Cost (₹ INR)	Wastage Disposal Cost	Cost of Water	Total Coolant Cost (₹)	Operator/ Environment Safety	Performance
1	Synthetic oil	MQL	220	225	49,500	Nil	Nil	49,500	Excellent	Excellent
2	Vegetable oil	MQL	188	225	42,300	Nil	Nil	42,300	excellent	Good
3	Waste oil	MQL	50	225	11,250	Nil	Nil	11,250	Moderate	Moderate
4	Mineral oil	MQL	228	260	59,280	Nil	Nil	59,280	Good	Moderate
5	Mineral oil	Flood	228	260	59,280	54,000	Yes	113,280	Less	Good
6	Cryogenic cooling	LN_2_	80–120	112,320	8,985,600	Nil	Nil	8,985,600	Good	Excellent
7	(LN_2_ + Vegetable oil)	MQL+ Cryo	140 (avg)	375	52,500	Nil	Nil	52,500	Good	Excellent
8	Nil	Dry	Nil	Nil	Nil	Nil	Nil	NA	Good	Poor
9	Vegetable oil + nano fluid	Hybrid NMQL	190 ₹ V.O and 4500 ₹ for (100 gm of N.F)	94 L (V.O), 2.82 kg (N.F)	17,860 (V.O), 126,900 (N.F)	Nil	Nil	144,760	Good	Good

**Table 10:** Achievement of SDG-12 indices.

S.N	SDG-12 Indices	Description	Implementation in Present Study	Impact
1	12.2	Minimization of natural resource	Utility of vegetable oil/waste oil	Conservation of natural resources
2	12.4	Responsible management of chemical and waste	MQL machining	Reduction in health hazard as well as economic machining
3	12.5	Substantially reduced waste generation	MQL machining using V.O and W.O	Wastage minimization
4	12.6	Practice of Sustainable Production	Low environmental hazards, waste reduction, economic machining, elimination of wastage disposal cost	Better performance index, Sustainable Manufacturing
5	12.8	Understanding of Sustainable Life cycle	Utility of MQL, V.O and W.O	An approach to sustainable machining/ manufacturing
6	12-A	Support to developing countries for Sustainable consumption and production	Reduction in wastage disposal, recycling cost and environmental degradation	Conservation of natural resources, cleaner production
7	12-C	Bar on wasteful consumption	Non-utilization of petroleum based mineral oil	Conservation of natural resources, cleaner production

## Data Availability

No data were used to support this study.
